# Acupuncture Point “Hegu” (LI4) Is Close to the Vascular Branch from the Superficial Branch of the Radial Nerve

**DOI:** 10.1155/2019/6879076

**Published:** 2019-06-25

**Authors:** Kanae Umemoto, Munekazu Naito, Kaori Tano, Hayato Terayama, Taro Koike, Mika Ohmichi, Yusuke Ohmichi, Kou Sakabe, Takashi Nakano

**Affiliations:** ^1^Department of Anatomy, Division of Basic Medical Science, Tokai University School of Medicine, 143 Shimokasuya, Isehara, Kanagawa, Japan; ^2^Department of Anatomy, Aichi Medical University School of Medicine, 1-1 Yazakokarimata, Nagakute, Aichi, Japan; ^3^Department of Acupuncture and Moxibustion, Faculty of Health Science, Suzuka University of Medical Science, 1001-1 Kishioka, Suzuka, Mie, Japan; ^4^Department of Anatomy, Kansai Medical University, 2-5-1 Shinmachi, Hirakata, Osaka, Japan

## Abstract

The acupuncture point “Hegu” (LI4) has been used for treating peripheral circulatory failure, which is located in the area covered by the superficial branch of the radial nerve (SBRN). SBRN has branches reaching arteries, so-called vascular branches (VBs), which are thought to be involved in the arterial constriction. The distribution areas of the VBs from the SBRN have been reported, but the positional relationship between these distribution areas and the acupuncture points are not known. To examine the positional relationship between LI4 and VBs from the SBRN, forty hands were examined to assess the positional relationship between the acupuncture points “Erjian” (LI2), “Sanjian” (LI3), LI4, and “Yangxi” (LI5) in the Yangming Large Intestine Meridian of Hand, which are located in the area covered by SBRN, and the VBs from the SBRN. After the VBs were identified, the distances from the acupuncture points (LI2, LI3, LI4, and LI5) to the point where the VBs reached the radial artery or the first dorsal metacarpal artery were measured. VBs reaching the radial arteries were observed in all specimens. The mean distances from LI2, LI3, LI4, and LI5 to the point where the VBs reached the radial artery were 64.2 ± 8.2 mm, 42.0 ± 7.5 mm, 4.3 ± 4.3 mm, and 33.0 ± 4.8 mm, respectively. LI4 was significantly closer than the other acupuncture points (P<0.01). The nerve fibers of the VBs adjacent to the radial artery were confirmed using hematoxylin and eosin staining. Our findings provide anatomical evidence that stimulation at LI4 is used for treating peripheral circulatory failure such as Raynaud's disease. LI4 is significant because it is located at a source point, making it clinically important.

## 1. Introduction

It has been reported that acupuncture increases skin blood flow [1]; therefore, stimulation at acupuncture points has been used for treating peripheral circulatory failure. “Hegu” (LI4) in the Yangming Large Intestine Meridian of Hand, a special acupuncture source point, has been used as an acupuncture point for treating peripheral circulatory failure in the hand. LI4 is located in the area covered by the superficial branch of the radial nerve (SBRN), and close to the radial artery or first dorsal metacarpal artery, which is a branch of the radial artery.

Cutaneous nerves have branches reaching arteries, the so-called vascular branches (VBs) [2–8]. Mogan and Balogh et al. found sympathetic fibers in the VBs from the cutaneous branch of the ulnar nerve and in the adventitia of the ulnar artery [6, 8]. Therefore, the VBs from the cutaneous nerves could be involved in the constriction of peripheral arteries. We previously reported that the distribution areas of the VBs from the SBRN were more limited than those of other nerves (i.e., lateral antebrachial cutaneous nerve, medial antebrachial cutaneous nerve, and cutaneous branch of the ulnar nerve) [9]. However, the positional relationship between the distribution areas of VBs from SBRN and LI4 has not been clarified.

In this study, we investigated the positional relationship between the acupuncture point LI4 and the distribution areas of VBs from the SBRN and confirmed the nerve fibers of the VBs adjacent to the radial artery using hematoxylin and eosin staining. We also discuss the anatomical evidence for why LI4 has been frequently used in cases of peripheral circulatory failure.

## 2. Methods

### 2.1. Anatomic Dissection

Twenty-seven human cadavers (11 males, 16 females) were examined in this study. The cadavers were donated to the Aichi Medical University School of Medicine during 2015 and 2016. Before death, the donors had given informed consent for the use of their bodies in clinical research. The format of the document is within the purview of the Japanese law “Act on Body Donation for Medical and Dental Education.” The Ethics Committee in Aichi Medical University School of Medicine approved this study (2016-M019). Cadavers with vascular grafts or fixed flexion in the hands were excluded because the pathways and distributions of VBs could not be confirmed accurately. Forty hands (18 males, 22 females) were examined to assess the positional relationship between the acupuncture points “Erjian” (LI2), “Sanjian” (LI3), LI4, and “Yangxi” (LI5) in the Yangming Large Intestine Meridian of Hand and VBs from the SBRN. The mean age of the cadavers was 85.4 ± 9.1 years (range, 69–104 years; males, 84.6 ± 8.1 years; females, 86.0 ± 9.9 years). All cadavers were embalmed using 10% formaldehyde with 4% phenol injection solution [9].

**Figure 1:**
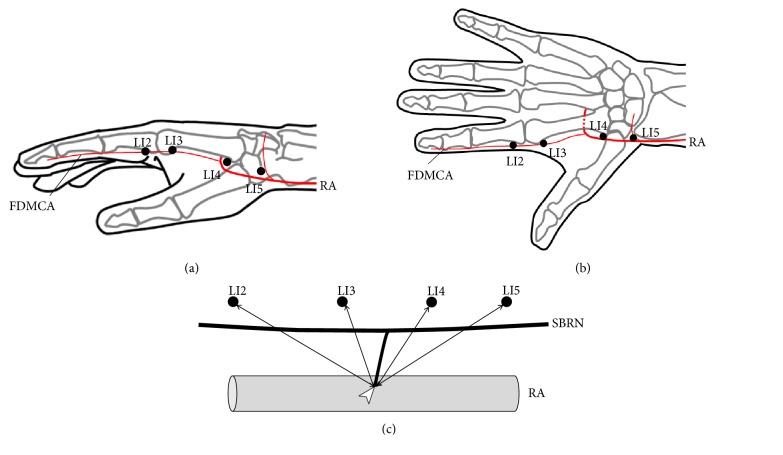
*Diagrams of anatomical measurements.* (a) Schematic of acupuncture points in the Yangming Large Intestine Meridian of Hand on the radial side. (b) Schematic of acupuncture points in the Yangming Large Intestine Meridian of Hand on the dorsal side. (c) Method for measuring vascular branches. LI2, acupuncture point “Erjian”; LI3, acupuncture point “Sanjian”; LI4, acupuncture point “Hegu”; LI5, acupuncture point “Yangxi”; RA, radial artery; FDMCA, first dorsal metacarpal artery; SBRN, superficial branch of radial nerve. White arrowheads indicate the points where the VBs reached the arteries.

Initially, LI2 was identified in a depression located on the radial side of the second finger distal to the second metacarpophalangeal joint. LI3 was identified on the radial side of the second finger, proximal to the head of the second metacarpal bone. LI4 was identified at the base of the first and second metacarpal bones on the dorsum. LI5 was identified on the radial side of the wrist in a depression between the extensor pollicis longus and brevis tendons (i.e., snuff box) (Figures 1(a) and 1(b)).

Next, the SBRN was identified after removal of the skin and subcutaneous tissue. The SBRN was identified as it exited the fascial layer between the tendon of the brachioradialis and the belly of the extensor carpi radialis longus muscle. The SBRN was followed distally to identify the points where the VBs reached the radial arteries or first dorsal metacarpal arteries [9]. After each hand had been marked at the correct anatomical positions, the distances from the acupuncture points to the points where the VBs reached the arteries were measured (Figure 1(c)). All measurements were made using digital calipers, to the nearest 0.1 mm. SPSS version 22.0 (IBM Corp., Armonk, NY, USA) was used for statistical analyses [9]. The distances from the acupuncture points to the points where the VBs reached the arteries were compared among acupuncture points using the Dunnett test. Results are presented as mean ± standard deviation (SD). The threshold of statistical significance was set at P<0.05.

### 2.2. Tissue Sampling

VBs with the radial artery were obtained from dissected human cadaveric hands. The collected samples were fixed with labels in 10% neutral buffered formalin (4% formaldehyde phosphate buffered saline) and processed as per standard protocol. Paraffin embedding of the collected samples was done after dehydrating the fixed tissue in ascending grades of alcohol solution. Xylene, which is miscible with both alcohol and paraffin, was used to remove the alcohol. The blocks were made by using melted paraffin that was allowed to cool. Once hardened, they were cut to an appropriate size. Sectioning of the tissue samples was done by rotary microtome. Every 5th section (3-*μ*m thick) was obtained and stained with hematoxylin and eosin. Samples were immersed in xylene and alcohol, stained with hematoxylin for 15 min, stained with eosin for 1 min, and then reimmersed in alcohol and xylene. Slides were mounted using a synthetic resin. Areas along the blood vessels were observed and photographed with an Olympus BX63 automated microscope (Olympus, Tokyo, Japan), PlanApoN x 2/0.08 objective lens (Olympus, Tokyo, Japan), UPlanSApo × 4/0.16 objective lens (Olympus, Tokyo, Japan), and Olympus DP73 CCD digital camera (Olympus, Tokyo, Japan).

## 3. Results

VBs from the SBRN in 40 of the 40 specimens reached the radial artery. After removal of the skin and subcutaneous tissue, we confirmed the paths of the radial artery and the SBRN. After the radial artery reaches the snuff box, the radial artery passes from the back of the hand into the palm between the two heads of the first dorsal interosseous muscle, the radial artery branches off the first dorsal metacarpal artery.

**Figure 2:**
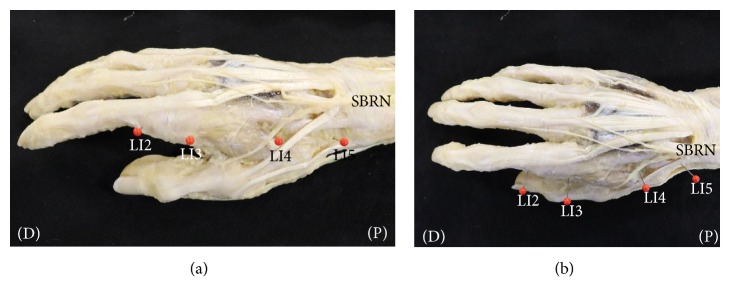
*Dissection photographs showing the acupuncture points in the Yangming Large Intestine Meridian of Hand and the distribution of the superficial branch of the radial nerve (SBRN) on the right side.* (a) Dissection photographs on the radial side. (b) Dissection photographs on the dorsal side; LI2, acupuncture point “Erjian”; LI3, acupuncture point “Sanjian”; LI4, acupuncture point “Hegu”; LI5, acupuncture point “Yangxi”; SBRN, superficial branch of radial nerve; (P), proximal (D), distal. The acupuncture points in the Yangming Large Intestine Meridian of Hand and the SBRN were identified after removal of the skin and subcutaneous tissue.

**Figure 3:**
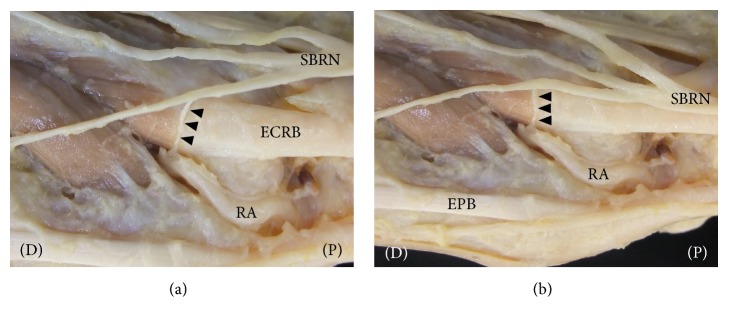
*Dissection photographs showing the vascular branches (VBs) of the superficial branch of the radial nerve (SBRN) on the right side.* (a) Dissection photographs on the radial side after removal of the skin and subcutaneous tissue. (b) Dissection photographs on the dorsal side after removal of the skin and subcutaneous tissue. RA, radial artery; SBRN, superficial branch of radial nerve; ECRB, extensor carpi radialis brevis tendon; EPB, extensor pollicis brevis muscle. Black arrowheads indicate the vascular branches (VBs). (P), proximal (D), distal.

After the SBRN exits the fascial layer between the tendon of the brachioradialis and the belly of the extensor carpi radialis longus muscle, the SBRN is distributed along the first, second, and third fingers on the radial side. We identified the pathway of the SBRN and LI2, LI3, LI4, and LI5 after removal of the skin and subcutaneous tissue. The SBRN in 40 of the 40 specimens ran along the second finger on the radial side (i.e., the pathway of the Yangming Large Intestine Meridian of Hand). LI2, LI3, LI4, and LI5 were close to the pathway of the SBRN (Figures 2(a) and 2(b)). The radial artery, running through LI4 in 40 of the 40 specimens, was distributed in the VBs of the SBRN (Figures 3(a) and 3(b)).

**Figure 4:**
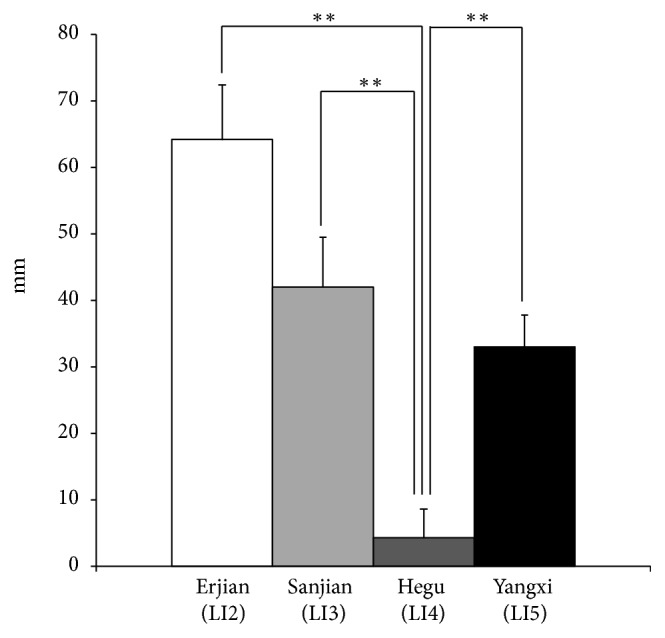
*The mean distances between the points where the VBs reached the radial artery and the positions of acupuncture points in the Yangming Large Intestine Meridian of Hand.* LI4 was significantly closer to the point where the VB reached the radial artery than were LI2, LI3, and LI5 (Dunnett test, P<0.01). *∗∗*P < 0.01, vs. LI4. Bars represent means with standard deviations (SD).

**Figure 5:**
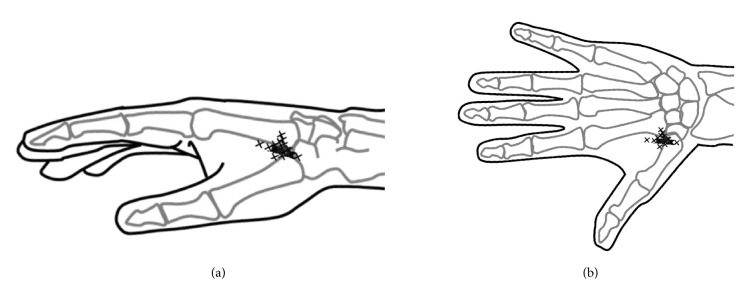
*Diagrams showing the distribution of the points where the VBs reached the radial artery.* (a) Schematic of the distribution of the points where the VBs reached the radial artery on the radial side. (b) Schematic of the distribution of the points where the VBs reached the radial artery on the dorsal side. Positions marked with an X indicate the points where the VBs reached the radial artery.

**Figure 6:**
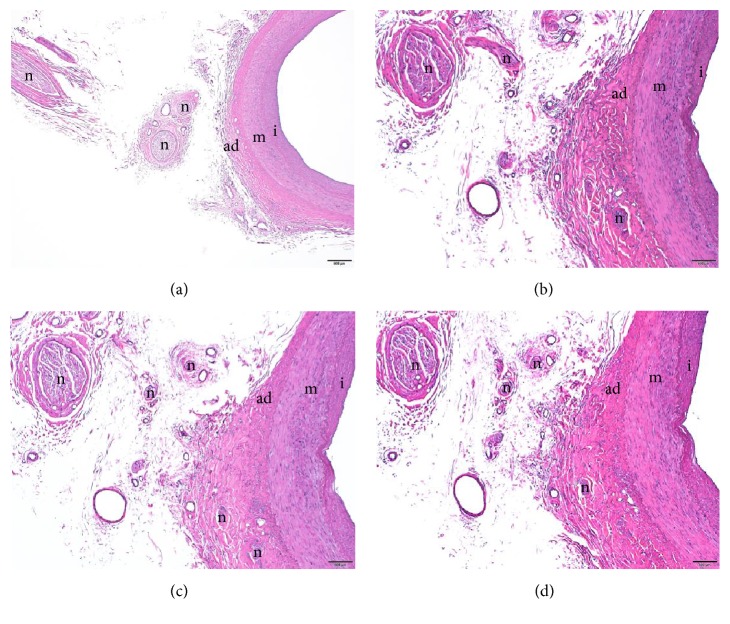
*A vascular branch of the superficial branch of the radial nerve (SBRN).* The nerve fibers of the vascular branch were adjacent to the radial artery (hematoxylin eosin stain). m, media; ad, adventitia; i, intima; n, nerve fibers of the vascular branch. All scale bars indicate 500 *μ*m (Panel (a) ×2/0.16 objective lens; Panel (b)-(d) ×4/0.16 objective lens).

The mean distances from LI2, LI3, LI4, and LI5 to the point where the VBs reached the radial artery were 64.2 ± 8.2 mm, 42.0 ± 7.5 mm, 4.3 ± 4.3 mm, and 33.0 ± 4.8 mm, respectively (Figures 4, 5(a), and 5(b)). LI4 was significantly closer to the point where the VB reached the radial artery than LI2, LI3, and LI5 (Dunnett test, P < 0.01) (Figures 4, 5(a), and 5(b)). The nerve fibers of the VBs were adjacent to the radial artery (Figure 6).

## 4. Discussion

In this study, we measured the distances from LI2, LI3, LI4, and LI5 to the points where the VBs reached the radial artery or first dorsal metacarpal artery; we found that LI4 was the closest.

The acupuncture points of the Yangming Large Intestine Meridian of Hand are used for stiff shoulders, numbness, and arthritis of the fingers, toothache, sore throat, eye pain, and constipation [10]. In addition, the acupuncture points are used for treating peripheral circulatory failure. LI2 and LI3 are close to the first dorsal metacarpal artery, which is a branch of the radial artery, and LI4 and LI5 are close to the radial artery.

Our findings confirmed that the SBRN runs along the second finger on the radial side. Therefore, we speculated the same effect could be achieved by acupuncture throughout LI2-LI5. However, LI4, a special acupuncture point as a source point, is used for a range of treatments [10]. The source point is the reactive point that appears when one or more of the five viscera are affected; therefore, this acupuncture point is used very extensively in the clinical setting [11]. For many years, the source point has been considered as a special anatomical site; however, the reasons for this were not clear. This study revealed that LI4 was significantly closer to the point where the VB reached the radial artery than LI2, LI3, or LI5 (Dunnett test, P< 0.01) (Figure 4), with a mean distance of 4.3 ± 4.3 mm (Figures 4, 5(a), and 5(b)) and confirmed histologically that the nerve fibers of the VBs were adjacent to the radial artery (Figure 6).

It is well known that stimulation at an acupuncture point can increase skin blood flow. Donoyama et al. reported that the skin temperature of the finger tips in a patient with Raynaud's disease increased after acupuncture treatment. In addition, the nail color, which changes from white to red with Raynaud's disease, returned to normal after acupuncture treatment [12]. Appiah et al. suggested that traditional acupuncture is a reasonable alternative for treating patients with Raynaud's disease [13]. Therefore, acupuncture may offer a potential cure for Raynaud's disease. A surgical treatment, periarterial sympathectomy, in which the adventitia surrounding the arteries in the hand and forearms innervated by VBs are stripped off, is used in cases of intractable Raynaud's disease [14–16]. Based on our findings, we suggest that LI4 may be the most suitable site for the adventitia to be stripped off in periarterial sympathectomy surgery. Additionally, acupuncture in this location (within a range of 4.3 ± 4.3 mm from the artery) can be effective for the peripheral circulatory failure that occurs in Raynaud's disease.

Substance P (SP) and calcitonin gene-related peptide (CGRP) are reported to be expressed at higher levels in the cutaneous nerve fibers of LI4 after stimulation at LI4 when compared with its expression in unstimulated rats [17]. Toma et al. reported that stimulation at LI4 attenuated when skin sympathetic nerve activity (SSNA) increased, although stimulation at LI4 did not affect the resting SSNA in healthy subjects [18]. SP and CGRP and decreased SSNA are known to cause the local blood vessels to dilate, which causes an increase in blood flow, causing warming. Stimulation at LI4 in healthy subjects produces a warming effect [19]. Omole et al. reported that stimulation at LI4 for 5 min twice weekly for 2 months resulted in improvements in pain severity, joint stiffness, and the color of the fingers and toes [20]. These findings are also evidence that acupuncture at LI4 can improve skin blood flow and be used to treat Raynaud's disease. Because our findings revealed that LI4 is the suitable site for stripping off adventitia in periarterial sympathectomy surgery, we presume that stimulation at LI4 can inhibit sympathetic nerve activity and improve Raynaud's disease.

Periarterial sympathectomy for the treatment for Raynaud's disease can cause patient anxiety and economic burden. Alternatively, because acupuncture is noninvasive and of low cost, patient anxiety and economic burden may be reduced. In this study, we suggested that LI4 is the suitable site for adventitia removal in periarterial sympathectomy surgery. Therefore, we presume that stimulation at LI4 is effective for the treatment of peripheral circulatory failure that occurs in Raynaud's disease.

## Data Availability

The data used to support the findings of this study are available from the corresponding author upon request.
